# *Streptococcus pneumoniae* Clonal Complex 199: Genetic Diversity and Tissue-Specific Virulence

**DOI:** 10.1371/journal.pone.0018649

**Published:** 2011-04-14

**Authors:** Jonathan C. Thomas, Marisol Figueira, Kristopher P. Fennie, Alison S. Laufer, Yong Kong, Michael E. Pichichero, Stephen I. Pelton, Melinda M. Pettigrew

**Affiliations:** 1 Division of Epidemiology of Microbial Diseases, Yale School of Public Health, Yale University School of Medicine, New Haven, Connecticut, United States of America; 2 Department of Microbiology, University of Mississippi Medical Center, Jackson, Mississippi, United States of America; 3 Boston University School of Medicine and Public Health, Boston Medical Center, Boston, Massachusetts, United States of America; 4 Yale University School of Nursing, New Haven, Connecticut, United States of America; 5 Department of Molecular Biophysics and Biochemistry, W. M. Keck Foundation Biotechnology Resource Laboratory, New Haven, Connecticut, United States of America; 6 Rochester General Hospital Research Institute, Rochester, New York, United States of America; National Institute of Allergy and Infectious Diseases, National Institutes of Health, United States of America

## Abstract

*Streptococcus pneumoniae* is an important cause of otitis media and invasive disease. Since introduction of the heptavalent pneumococcal conjugate vaccine, there has been an increase in replacement disease due to serotype 19A clonal complex (CC)199 isolates. The goals of this study were to 1) describe genetic diversity among nineteen CC199 isolates from carriage, middle ear, blood, and cerebrospinal fluid, 2) compare CC199 19A (n = 3) and 15B/C (n = 2) isolates in the chinchilla model for pneumococcal disease, and 3) identify accessory genes associated with tissue-specific disease among a larger collection of *S. pneumoniae* isolates. CC199 isolates were analyzed by comparative genome hybridization. One hundred and twenty-seven genes were variably present. The CC199 phylogeny split into two main clades, one comprised predominantly of carriage isolates and another of disease isolates. Ability to colonize and cause disease did not differ by serotype in the chinchilla model. However, isolates from the disease clade were associated with faster time to bacteremia compared to carriage clade isolates. One 19A isolate exhibited hypervirulence. Twelve tissue-specific genes/regions were identified by correspondence analysis. After screening a diverse collection of 326 isolates, spr0282 was associated with carriage. Four genes/regions, SP0163, SP0463, SPN05002 and RD8a were associated with middle ear isolates. SPN05002 also associated with blood and CSF, while RD8a associated with blood isolates. The hypervirulent isolate's genome was sequenced using the Solexa paired-end sequencing platform and compared to that of a reference serotype 19A isolate, revealing the presence of a novel 20 kb region with sequence similarity to bacteriophage genes. Genetic factors other than serotype may modulate virulence potential in CC199. These studies have implications for the long-term effectiveness of conjugate vaccines. Ideally, future vaccines would target common proteins to effectively reduce carriage and disease in the vaccinated population.

## Introduction

*Streptococcus pneumoniae* asymptomatically colonizes the upper respiratory tract of approximately half of all healthy children and is a leading cause of acute otitis media, pneumonia and meningitis globally [1]–[3]. Although our comprehension of the epidemiology, pathogenesis, and virulence factors of *S. pneumoniae* has improved in recent years, the basis for whether colonization with a specific strain establishes asymptomatic colonization or produces local or invasive diseases requires further elucidation.

Encapsulated strains of *S. pneumoniae* express one of at least 93 distinct capsular polysaccharides [4], [5]. Since the introduction of the heptavalent pneumococcal conjugate vaccine (PCV7; serotypes 4, 6B, 9V, 14, 18C, 19F and 23F) in 2000, a large overall decrease in invasive disease continues to be observed [6], [7]. However, non-vaccine serotypes have increased in prevalence [8]–[11]. Studies show a significant increase in the number of otitis media and invasive disease cases due to serotype 19A [8], [12]–[14]. In the United States and Europe, clonal complex (CC)199 was a major clonal lineage throughout the period of expansion of serotype 19A [9], [14]–[17]. A small percentage of these CC199 19A isolates are associated with antimicrobial resistance [14]. CC199 strains may also express the 15B/C capsule. Serogroup 15 isolates have recently increased in prevalence, albeit to a lesser extent than serotype 19A [18], [19]. Serotype 19A is now included in the expanded pneumococcal conjugate vaccine (PCV13), while serotype 15B/C is not.

Differences in virulence have been reported between pneumococcal serotypes [11], [20]–[22]. Furthermore, differences in virulence within a serotype have been reported in animal models [23] and population based studies [24]. Even closely related isolates belonging to the same clone or sequence type (ST) can differ in their capacity to cause disease [23].

The pneumococcal genome exhibits high plasticity and may be categorized into the core genome, consisting of genes conserved between all *S. pneumoniae* isolates, and the accessory genome, consisting of genes that are variably present throughout the *S. pneumoniae* population [25]–[27]. Between 21–32% of genes in a given strain belong to the accessory genome [26]. It has been hypothesized that some genes may engender tissue-specific advantages [23], [28], such as providing the isolate with an increase in fitness or capacity to invade a given niche. In the identification of tissue-specific genes, studies have often focussed on established pneumococcal virulence factors or genes that are differentially expressed *in vivo* [29]–[31]. Few studies have examined the non-core component of the pneumococcal genome to identify genes that provide a biological basis for tissue-specificity. Comparative genome studies have focussed on invasive disease rather than otitis media as a disease outcome [23], [27], [32].

This study sought to evaluate genetic variation among CC199 isolates, and to identify genes associated with strains isolated from a particular tissue source. CC199 isolates of the same genetic background but different serotype (serotype 19A and 15B/C) were compared in the chinchilla model of pneumococcal disease to assess the relationship between genetic diversity and capsular serotype on virulence. Importantly, this model allowed us to ascertain virulence for otitis media. These strains also produced bloodstream infection, permitting assessment of relative virulence for invasive disease. The initial analysis of related isolates from the same clonal complex reduced the genetic variation identified, and consequently the level of noise encountered while identifying tissue-associated genes among CC199. To gain additional insight into *S. pneumoniae* tissue-associated genes, we identified genetic regions associated with specific tissue sources among CC199 isolates and used these to screen a larger, genetically diverse collection of pneumococcal isolates. We reasoned that if the identified genes were truly important for tissue-specific virulence, then they would also occur more frequently among diverse pneumococcal sequence types and serotypes from these tissue sites.

## Results

### Genomic Diversity within Clonal Complex 199

Comparative genome hybridization (CGH) was used to evaluate genetic diversity among nineteen CC199 isolates. One hundred and twenty seven genes were found to be variable within CC199 (Table S1). The CGH results identified four regions of diversity (RD), using the criteria specified by previous studies investigating pneumococcal genomic diversity [23]. These included RD2, RD6, and RD24 [23], and RD8 [27] (Table 1). The regions of diversity differed from those previously described by Silva *et al.* RD2 identified within CC199 isolates did not contain SP0114 and SP0115 and was approximately 0.7 kb smaller; RD24, identified within CC199 isolates, was slightly larger due to the addition of SP1947.

**Table 1:** Regions of genetic diversity identified in clonal complex 199 isolates.

Region of	Genes	Size*	Encodes§
Diversity			
2	SP0109	∼4.2 kb	Putative Bacteriocin
	-		Putative Amino Acid ABC Transporters
	SP0113		Hypothetical Protein
6	SP0394	∼5.3 kb	Putative transcriptional regualtor
	-		Mannitol Phosphate Dehydrogenase
	SP0397		Phophotransferase System
8	SP1315	∼31.5 kb	Sodium ATP Synthase
	-		Oxidoreductase
	SP1351		Putative neuraminidase
			N-Acetylneuraminate Lyase
			Putative N-Acetylmannosamine-6-P Epimerase
			Putative Phosphosugar-Binding Transcriptional Regulator
			Methyltransferase
			Transposase
			ABC Transporter/ATP Binding Protein
			Drug Efflux ABC Transporter
			Prolyl Oligopeptidase Family Protein
			Putative Membrane Protein
			Hypothetical Proteins
24	SP1947	∼9.0 kb	Putative Bacteriocin Formation Protein
	-		Toxin Secretion ABC Transporter
	SP1955		Serine Protease
			Hypothetical Proteins

*Based on gene sizes taken from TIGR4 genome.

§ Based on annotation taken from TIGR4 genome.

**Figure 1:**
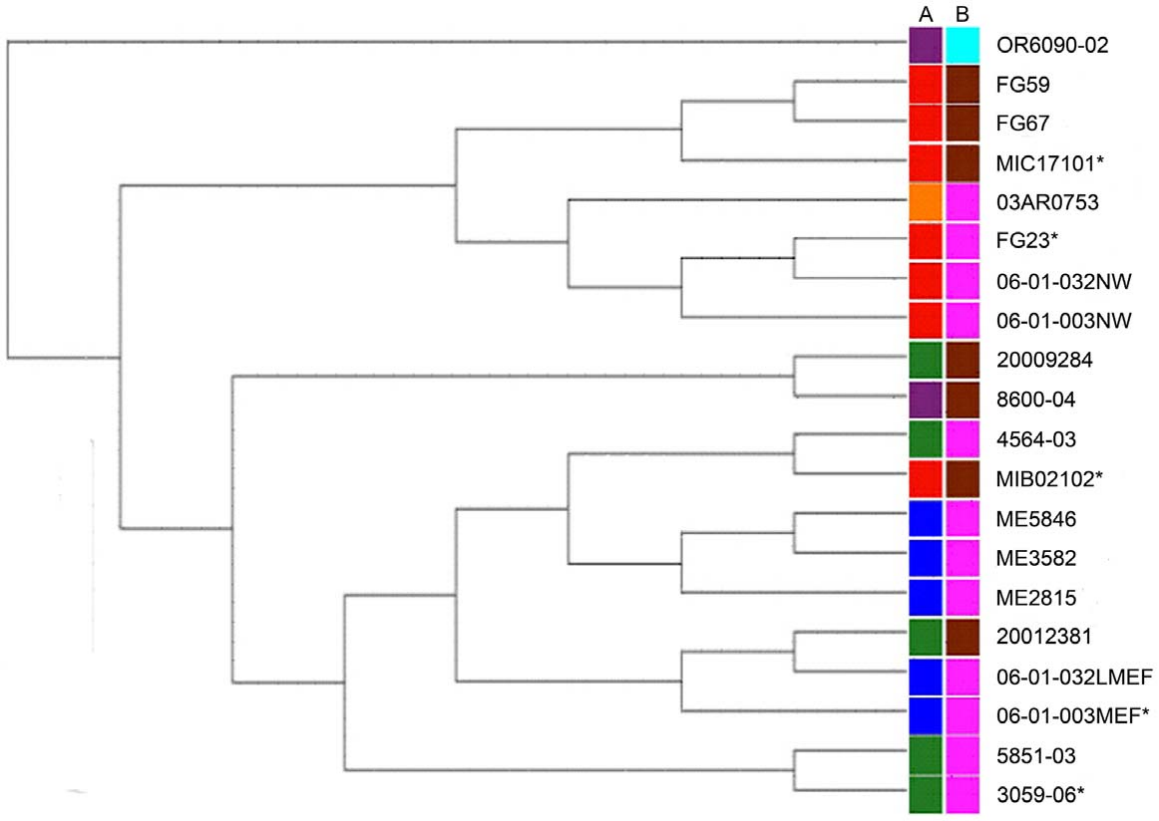
Hierarchical clustering dendrogram of clonal complex 199 isolates. *Strain tested in the chinchilla model of disease Coloured column one relates to tissue source. Red = carriage, Blue = Middle ear fluid, Green = Blood, Purple = Cerebrospinal fluid, Orange = Pleural Fluid. Coloured column two relates to serotype. Bright blue = 3, Brown = 15B/C, Pink = 19A.

A phylogeny of the isolates was constructed by hierarchical clustering based on the log_2_ ratio matrix (Figure 1). The phylogeny obtained by hierarchical clustering closely agreed with the dendrogram obtained by Dollo parsimony of the presence and absence matrix (data not shown). CC199 splits into two main clades, one of which consists of all but one of the disease isolates, while the other comprises all but one of the carriage isolates (Figure 1). The carriage clade is further split, based on serotype, while the disease clade exhibits no additional structure based on serotype.

### Chinchilla Model of Pneumococcal Disease

**Figure 2:**
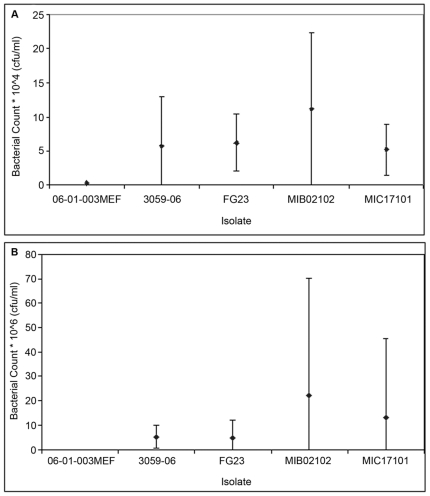
Bacterial counts for each of the five CC199 pneumococcal isolates tested in the chinchilla model of pneumococcal disease. A. Bacterial counts from nasopharyngeal washes obtained on day 1. B. Bacterial counts from middle ear fluid samples obtained on day 8.

We next sought to compare the virulence of carriage and disease clade isolates in the chinchilla model of disease. Five representative CC199 strains were selected for testing. In addition to a genome sequenced reference 19A strain (CDC3059-06), one serotype 15B/C and one serotype 19A isolate were selected from both the carriage and invasive clades (Figure 1). None of the CC199 isolates exhibited a significantly increased ability to colonize (Figure 2a) or cause otitis media (Figure 2b). There was little difference between serotype 19A and serotype 15B/C isolates' ability to cause otitis media (Figure 2b) or in the time taken for the isolate to cause bacteremia (p = 0.63).

While the isolates did not differ in their virulence potential by serotype, isolates belonging to the disease clade of the CC199 phylogeny did exhibit a decreased duration of colonization before causing bacteremia compared to carriage clade isolates (p = 0.04). Of the isolates from the disease clade, one serotype 19A isolate (06-01-003 MEF) demonstrated an increased invasive virulence potential beyond that of the other isolates, resulting in chinchilla mortality within 48–72 hours, significantly more rapidly than other disease isolates (p<0.0001).

### Tissue-Specific Genes

The separation of the phylogeny of CC199 based predominantly on isolate tissue source (i.e. carriage vs. disease), combined with the similar fitness of isolates by serotype, indicates that genetic factors other than serotype impact on fitness for tissue-specific virulence. The microarray analysis was not able to identify any single genetic factor as being responsible for the difference between the carriage and disease clades. Therefore, we sought to identify non-core genes that might influence tissue-specific virulence.

Correspondence analysis identified 11 genes as well as the region of diversity RD8a as being associated with CC199 isolates obtained from a particular tissue source. Next, we screened a larger, genetically diverse collection of *S. pneumoniae* isolates (n = 326). Eight of the 11 genes were significantly associated with a specific tissue source after screening of the larger collection. Two of these genes encoded choline-binding proteins (*cbpC* and *cbpJ*), which were negatively associated with invasive strains, compared to carriage, as determined by PCR (OR [95% CI]): 0.46 [0.23, 0.90] and 0.33 [0.17, 0.61] for *cbpC* and *cbpJ*, respectively. These findings could not be confirmed by hybridization due to the cross-hybridization of the probe with other choline binding protein-encoding genes in the genome.

Based on PCR, dot blot, and Southern hybridization screening, seven genetic regions were found to be significant in their association with either a particular tissue source, or with either invasive or non-invasive isolates. The prevalence data for the seven genetic regions within CC199 are in Table 2. It should be noted that correspondence analysis does not provide a statistical test of the strength of the association and a limited number of CC199 strains were examined. Among the larger collection of *S. pneumoniae* isolates, the prevalence ranged from 29% (RD8 in carriage isolates) to 81% (SP1793 in CSF isolates) (Table 2). The odds ratios and 95% CI for each genetic region by tissue source are presented in Table 3. SPN05002, which encodes a hypothetical protein, was present significantly more frequently in strains isolated from middle ear fluid, blood, and CSF than from those strains isolated from carriage. The genetic region RD8a was also present significantly more frequently in strains isolated from middle ear fluid and blood than carriage. Conversely, spr0282, which encodes a phosphotransferase system sugar-specific EII component, was present significantly more frequently in strains isolated from carriage than from middle ear fluid or blood.

**Table 2:** Distribution of putative tissue-specific genes among *S. pneumoniae* isolate collections.

	No. of isolates positive for given gene (%)						
Collection	SP0163	SP0395	SP0463	SP1793	SPN05002	spr0282	RD8a
**CC199**							
Carriage (n = 7)	6 (86)	3 (43)	2 (29)	3 (43)	4 (57)	0 (0)	7 (100)
Middle Ear (n = 5 )	3 (60)	2 (40)	3 (60)	3 (60)	1 (20)	0 (0)	1 (20)
Blood (n = 5)	4 (80)	1 (20)	3 (60)	4 (80)	2 (40)	1 (20)	2 (40)
**Larger Collection**							
Carriage (n = 92)	45 (49)	59 (64)	35 (38)	56 (61)	31 (34)	66 (72)	27 (29)
Middle Ear (n = 94)	68 (72)	43 (46)	58 (62)	53 (56)	60 (64)	50 (53)	48 (51)
Blood (n = 83)	44 (53)	56 (67)	39 (47)	52 (63)	60 (72)	46 (55)	41 (49)
CSF (n = 57)	27 (47)	44 (77)	19 (33)	46 (81)	44 (77)	34 (60)	25 (44)
Total (n = 326)	184 (56)	202 (62)	151 (46)	207 (63)	195 (60)	196 (60)	141 (43)

*RD8 was present in the CSF isolate. SP0163, SP0395, spr0282, and RD8a were present in the pleural fluid isolate.

**Table 3:** Adjusted odds ratios for each of the tissue-specific genes identified by correspondence analysis.

Locus	Product	Odds Ratios				
		Middle ear vs Carriage	Blood vs Carriage	CSF vs Carriage	Disease vs Carriage	Invasive vs Non
SP0163	Transcriptional Regulator	**2.73 (1.48, 5.02)**	1.18 (0.65, 2.13)	0.94 (0.48, 1.82)	1.53 (0.94, 2.48)	0.66 (0.43, 1.04)
SP0395	Transcriptional Regulator	**0.45 (0.25, 0.82)**	1.23 (0.65, 2.30)	1.89 (0.89, 4.01)	0.88 (0.53, 1.44)	**2.18 (1.37, 3.49)**
SP0463SP1793	Cell Wall Surface Anchor ProteinHypothetical Protein	**2.49 (1.38, 4.47)**0.83 (0.46, 1.49)	1.44 (0.79, 2.64)1.08 (0.59, 1.99)	0.81 (0.41, 1.63)**2.69 (1.23, 5.86)**	1.57 (0.96, 2.58)1.24 (0.76, 2.05)	0.72 (0.46, 1.12)**1.65 (1.04, 2.62)**
SPN05002	Hypothetical Protein	**3.13 (1.72, 5.67)**	**4.89 (2.57, 9.32)**	**6.35 (2.99, 13.47)**	**4.27 (2.56, 7.12)**	**3.01 (1.88, 4.85)**
spr0282	Phosphotransferase System	**0.43 (0.23, 0.78)**	**0.49 (0.26, 0.92)**	0.58 (0.29, 1.17)	**0.48 (0.29, 0.81)**	**0.51 (0.33, 0.79)**
RD8a	Region of Diversity	**2.41 (1.32, 4.39)**	**2.35 (1.26, 4.38)**	1.88 (0.94, 3.75)	**2.25 (1.34, 3.77)**	1.34 (0.86, 2.09)

Odds ratios in bold indicate those that were significant.

### Hypervirulent Isolate

As described above, one isolate 06-01-003MEF (CC199, 19A) was found to have an increased invasive virulence potential in the chinchilla model. No single genetic variation could be found by microarray to explain the increased virulence observed for the serotype 19A isolate. While current microarray slides comprise probes for genes from several completely sequenced genomes, the high degree of plasticity in *S. pneumoniae* genomes is such that microarrays will not possess the full complement of probes for a given isolate being tested. Therefore, a comparison was made of the genome sequences of the hypervirulent isolate and the isolate CDC3059-06 (CC199, 19A), which was representative of the virulence potential of the CC199 isolates in this study.

The genome of the hypervirulent isolate 06-01-003MEF was found to contain approximately 35 kb of genetic material not possessed by the reference isolate CDC3059-06, while CDC3059-06 contained approximately 42 kb of sequence not found in the genome of 06-01-003MEF. The three remaining CC199 isolates tested in the chinchilla model of pneumococcal disease were screened for the presence or absence of these genetic regions, both by PCR and by examining the microarray data. A single genetic region, approximately 20 kb in size, was present in the genome of the hypervirulent isolate 06-01-003MEF, but absent in CDC3059-06 and all three of the other isolates tested in the chinchilla model of pneumococcal disease. The region comprised at least 25 predicted genes, with ∼65% of the sequence comprising coding sequence and a GC content of ∼39%. When queried against the NCBI BLAST database, 14 of the 25 predicted genes were of unknown function, while the remainder corresponded to bacteriophage structural genes.

## Discussion

The data presented in this study supports previous research that has identified *S. pneumoniae* as a genetically highly heterogeneous species, even within closely related strains [23], [26]. The ability to colonize and cause disease did not differ depending on the isolate's serotype when tested in the chinchilla model. However, isolates in the disease clade were associated with a decreased time to cause bacteremia compared to isolates in the carriage clade. One hypervirulent serotype 19A isolate was associated with a short time to bacteremia and high mortality. The genome of this strain contains a 20 kb genetic region that is absent in the other CC199 isolates tested in the chinchilla. This hypervirulent strain may have acquired a bacteriophage with novel virulence factors, although further analysis is required to identify the precise role of this sequence, if any, in the observed hypervirulence of isolate 06-01-003MEF. Taken together, these data indicate that genetic factors, other than capsule polysaccharide, modulate virulence potential in CC199. Serotype 15B/C is not included in PCV13 [32]. Given the similar genetic background and similar pathogenicity in the chinchilla, CC199 strains of serotype 15B/C may increasingly cause acute otitis media in children.

We hypothesize that CC199 strains are successful at the population level because they are a generalist lineage adept at carriage, otitis media, and invasive disease. Within CC199, isolates of both serotype 15B/C and 19A were equally able to colonize and cause otitis media in the chinchilla model. Furthermore, there was little difference in the time taken to cause invasive disease by serotype. These data expand upon a previous study that indicated similar fitness for otitis media when serotype 19A and 15B/C isolates were inoculated together in competition [34]. While isolates belonging to the carriage clade have the potential to cause disease, isolates from the disease clade were associated with faster development of invasive disease. It remains to be seen if the identified pattern of separate carriage and disease clades in CC199 is repeated for other established clones, such as CC156 or CC176.

This study has identified genes of potential importance in tissue-specific virulence, which will require additional study to assess their potential and function as virulence factors. A larger collection more representative of the pneumococcal population was screened to determine whether those associations identified in CC199 isolates would be maintained in a more genetically diverse collection. Given that *S. pneumoniae* is a highly recombinogenic species, tissue-specific associations that are preserved throughout the population as a whole would be expected to be of biological importance to the species. However, it is worth noting that for two of the genetic loci, spr0282 and RD8a, the tissue-specific associations differed in the overall pneumococcal population when compared to the CC199 data, although no statistical significance was given to the CC199 data due to small sample size. This would seem to indicate that the effect of a gene may be dependent on the combination of genes found in the remainder of the pneumococcal genome.

Previous studies have identified individual genes associated with otitis media, pneumonia, and meningitis [31], [35]–[37]. We identified RD8a as more common among our middle ear and blood isolates in comparison to carriage. Obert *et al* previously described the correlation between the region of diversity RD8a and invasive isolates [27]. SP0463 (*rrgB*) is on the pilus locus [38] and has previously been identified in a minority of invasive pneumococcal isolates [39]. Our finding that SP0463 is found significantly more often in middle ear isolates than carriage isolates seems at variance with other data that pneumococcal isolates do not contain genes encoding pili at a greater frequency in otitis media isolates than in invasive disease isolates [40]. It is likely that the disparity arises through the specific isolate collections examined or the difference in target genes used by each study. Moschioni *et al* [40] targeted entire pilus encoding islets, this study focussed on a single gene within the islet.

The specific combination of putative tissue-specific genes differed for each of the isolates tested in the chinchilla model. Furthermore, we did not identify a single genetic factor from the accessory genome, present in all strains from a specific tissue source. A supragenome pool exists for *S. pneumoniae*, which consists of the total number of genes available to the species [26], [41], [42]. A given gene may associate with *S. pneumoniae* isolates from a specific tissue-source. Furthermore, a given gene may enhance fitness for tissue-specific disease, yet an isolate lacking this gene can still cause disease. These data may be explained by high levels of recombination and functional redundancy amongst the accessory regions within the pneumococcal genome. Blomberg *et al* did not identify a unique pattern of accessory regions among invasive disease isolates and concluded that redundancy existed amongst the accessory regions required to cause invasive pneumococcal disease [32]. Our data indicate that this redundancy holds for otitis media as well as invasive disease.

One hypervirulent CC199 19A (06-01-003 MEF) strain caused rapid mortality. The bacteriophage sequence within this region may encode virulence determinants. A majority of *S. pneumoniae* strains contain lysogenic phage [43], [44]. Researchers have speculated that phage-encoded proteins modulate *S. pneumoniae* fitness [45]. Further analysis of this sequence is required to identify if this is indeed the basis for the differences in virulence observed between the isolates.

This study had limitations. Genomic diversity of CC199 was evaluated using CGH. As mentioned previously, microarrays are limited in that genetic regions that are not included on the array will be missed. Genes that are highly variable in sequence may also fail to hybridize and will be counted as absent. Additional unmeasured factors, such as level of capsule expression, may also contribute to differences in virulence. As with any animal model, our data may not be fully reflective of *S. pneumoniae* carriage and disease in humans. A small number of chinchillas were used in each experiment and the chinchilla model does not use a genetically pure line. However, the advantage to the chinchilla model is it closely mirrors pneumococcal disease aetiology where colonization is established before disease.

In summary, the approval of PCV13 by the FDA in 2010 [33] means that isolates belonging to serotype 19A should be protected against in the future. However, this expanded vaccine does not include protection against serotype 15B/C. Recent studies have indicated an increase in the prevalence of serogroup 15 isolates amongst carriage and otitis media [10], [18]. Small but significant increases in the proportion of serotype 15B/C isolates have been observed among invasive disease cases [7]. While immunization with PCV13 is likely to result in disease reduction, the similarities in terms of virulence between 19A and 15B/C isolates suggest that a vaccine based on genetic factors other than serotype is necessary, especially for otitis media and nonbacteremic pneumonia. Such genetic factors could belong to the core genome or comprise several from the accessory genome, which together cover the entire pneumococcal population. Alternatively, future vaccines could be targeted towards tissue-specific genes, allowing physicians to protect patients from invasive pneumococcal disease, while not affecting those pneumococcal isolates that exist among carriage isolates in the nasopharynx [46].

## Materials and Methods

### Ethics Statement

The Institutional Animal Care and Use Committee at Boston University Medical Center approved our animal care protocols as being consistent with humane treatment of laboratory animals and with standards set forth in the Guide for the Care and Use of Laboratory Animals and the Animal Welfare Act (Animal Welfare Assurance approval number A-3316-01). Thus, our study was carried out in strict accordance with the recommendations in the Guide for the Care and Use of Laboratory Animals of the National Institutes of Health. All efforts were made to minimize suffering. Procedures to obtain nasopharyngeal lavages, middle ear cultures and phlebotomy were performed under Ketamine and Xylazine anaesthesia. All animals were examined daily for activity levels, fluid intake, and temperature. Early euthanasia was used when labyrinthitis was present or when animals exhibited a decrease in weight greater than 10%. Animals were euthanized using CO_2_.

### Bacterial Isolates, Growth Conditions, and DNA Extraction

A collection of 20 *S. pneumoniae* isolates was selected for analysis by CGH. Nineteen CC199 isolates were selected from collections previously analyzed by multilocus sequence typing (MLST) [28], [47]. Isolates were obtained from a variety of tissue sources, including the nasopharynx or throat (n = 7), middle ear fluid (n = 5), blood (n = 5), cerebrospinal fluid (CSF) (n = 1), and pleural fluid (n = 1). CC199 isolates were serotype 19A (n = 7) or 15B/C (n = 12). A serotype 3 isolate (ST180) was included as an outgroup for phylogeny construction. All isolates were obtained from the United States. With only two exceptions, strains were isolated since 2000 (isolates ME2815 and ME3582 were isolated in 1996). Isolate 3059-06 came from a patient 47 years of age and isolate 03AR0753 came from a patient of unknown age. The remaining isolates were from patients less than 48 months of age. Isolates to be analyzed by CGH were grown overnight in Todd Hewitt, 5% yeast extract at 37°C with 5% CO_2_. DNA was extracted using QIAGEN 100/G genomic tips.

A larger genetically diverse collection of *S. pneumoniae* isolates [28], was screened to ascertain the prevalence of genes associated with CC199 isolates from a given tissue source. This genetically diverse collection was comprised of nasopharyngeal or throat isolates from healthy children (collectively analyzed as the “carriage group”) (n = 92), middle ear (n = 94), blood (n = 83), and cerebrospinal fluid (n = 57). *S. pneumoniae* strains represented a range of serotypes and sequence types. DNA extractions for isolates to be screened by PCR involved overnight growth on trypticase soy agar plates with 5% sheep blood and inoculation of a colony into a 96-well plate containing 50 µl of Tris-EDTA buffer followed by boiling for 10 min.

### DNA Labelling and Microarray Hybridization

DNA was labelled using a BioPrime® Plus Array CGH Indirect Genomic Labelling System (Invitrogen). DNA labelling and microarray hybridization was conducted by staff at the W.M. Keck Facility at Yale.

### Microarray Analysis

TM4: Microarray Software Suite (TIGR) [48] and SAS v9.1 (SAS Institute, Cary, NC) were used in data analyses. LOWESS (locally weighted scatterplot smoothing) was used to normalize raw data in a single experiment, with 50% of the data being used for smoothing. The LOWESS normalization was used as part of a pipeline in MIDAS, which includes total intensity normalization, LOWESS normalization, standard deviation regularization and low intensity filtering [48]. Hybridization spots with a signal to noise ratio of less than 2.0 were removed from further analysis. Partek® Genomics Suite™ 6.4 (Partek Inc., St. Louis, MO) was used to remove variation in the data due to batch effects.

Two different methods were applied to the microarray data to determine if a given gene was present or absent. The program GACK was used to convert the data from each microarray experiment to present or absent [49]. Alternatively, arbitrary cutoff values were selected. Hybridization spots with a log_2_ ratio above 1, 1.5, 2 or 2.5 were denoted as present, while those with a ratio below −1, −1.5, −2 or −2.5 were identified as absent. A range of cut-off value combinations were assessed.

Hierarchical clustering of the log_2_ ratio matrix, and visualization of the resulting dendrogram, was performed in Partek® Genomics Suite™ 6.4, using Pearson's Dissimilarity to calculate row dissimilarity and Ward's method for row clustering. A phylogeny of the isolates was also constructed using Dollo parsimony based on the presence and absence matrix of genes [50] implemented in PAUP4.0b10. Hybridization spots that produced missing data for more than 20% of the isolates were removed before phylogeny construction. Both dendrograms were rooted with a serotype 3, ST180 isolate.

All CGH data generated by this study were fully annotated and deposited at the Gene Expression Omnibus (GEO; Accession numbers GSM591013–GSM591034). Data uploaded to the GEO database is MIAME compliant, representing the final data from the normalisation pipeline. Subsequent data generated via batch effect removal could not be uploaded due to file formatting issues, but is available upon request.

### PCR Validation of Microarray Results

Eighteen genes, including both core and variable genes, were chosen to validate CGH results. Primer sequences were designed for each gene (Table S2). PCR involved an initial denaturation step of 95°C for 3 min; 35 cycles of 95°C for 30 sec, the relevant annealing temperature for 30 sec (Table S2), and 72°C for 30 sec; and a final extension of 72°C for 10 min.

### Chinchilla Model of Pneumococcal Disease

Five pneumococcal isolates were tested in the chinchilla model of pneumococcal disease. Female chinchillas (*Chinchilla laniger*) with no prior evidence of middle ear infection were used. One serotype 15B/C and one serotype 19A isolate were selected from each clade of the CC199 phylogeny. Isolates MIB02102 and MIC17101 were serotype 15B/C, and 06-01-003MEF and FG23 were serotype 19A, from the invasive and carriage clades, respectively. A genome sequenced reference strain (isolate CDC3059-06, serotype 19A) was also included.

Isolates were grown as monocultures and used to inoculate the left nare of two to eight healthy chinchillas [34], [51]. The *S. pneumoniae* strains were allowed to establish nasopharyngeal colonization. Nasopharyngeal washes were collected from all animals on day 1 and day 5 by lavage with Hanks buffer. Barotrauma, which creates negative pressure in the middle ear cavity by aspiration of up to 250 µl of air with a 25-guage needle, was performed unilaterally on day 5 to prompt development of otitis media. Chinchillas were monitored daily by otomicroscopy and tympanometry. Once the animal developed signs of otitis media, the middle ear cavity was accessed through a small hole in the bullar bone. Nasopharyngeal and middle ear samples were collected for quantitative microbiology. Total viable counts (CFU/ml) were obtained by plating on blood agar and incubation at 37°C overnight. Total viable counts were calculated from day 1, representing the initial colonization phase, and day 8, the first middle ear fluid sample obtained.

Differences in the total viable counts for each isolate were tested for by one-way analysis of covariance (ANCOVA), with Tukey, Bonferroni and Duncan post-tests being used for multiple comparisons. Weight of the individual chinchilla and the inoculation bacterial load were controlled for during these tests. The assumption of normality was not met. However non-parametric and parametric bivariate analyses provided similar results, suggesting the ANCOVA is robust enough to handle the violation of assumptions, and also allow us to control for weight and inoculation load. An isolate's propensity to cause invasive disease was determined through the length of time between colonization and the development of bacteremia. Differences in the time to bacteremia were tested using survival analysis (Kaplan-Meier survival curves, and log rank tests). The assumption of proportionality was met.

### Identification of Tissue-Specific Genes

Correspondence analysis was implemented in SAS v9.1 (SAS Institute, Cary, NC) to narrow the list of genes associated with isolates obtained from a particular tissue source (carriage, middle ear, or blood isolates). CSF and pleural fluid isolates were not included due to low numbers. For any given gene, its frequency amongst the isolates obtained from each tissue source was calculated, and used for each comparison. Correspondence analysis does not provide a measure of significance. Therefore the list of genes was verified and narrowed further by removal of genes that were equally present between tissue sources.

The larger isolate collection [29] was screened by PCR to ascertain the prevalence of genes identified as associated with a particular tissue source by correspondence analysis. Primers are detailed in Table S3. With the exception of primers to amplify *cbp*G [52], all primers were designed within this study. PCR involved an initial denaturation step of 95°C for 3 min; 35 cycles of 95°C for 30 sec, the relevant annealing temperature for 30 sec (Table S3), and 72°C for 30 sec; and a final extension of 72°C for 10 min. The presence or absence of each gene was confirmed for PCR-negative isolates by dot blot and Southern hybridization, using previously described methods [28]. Briefly, PCR amplification of TIGR4 genomic DNA was used to generate a gene-specific probe, using the gene-specific primer pairs listed in Table S3. The Gene Images AlkPhos Direct Labeling and ECF chemifluorescence detection system (Amersham Biosciences, Piscataway, N.J.) was used for labelling, hybridization, washes, and signal detection.

### Tissue-Specific Gene Analysis

Statistical analyses were carried out using SAS version 9.1 (SAS Institute, Cary, NC). The distribution of *S. pneumoniae* STs, clonal groups, and tissue-specific genes in the isolate collection were described using simple descriptive statistics. Logistic regression was used to calculate odds ratios and 95% confidence intervals for each gene for the isolate populations of each tissue source versus the carriage population. In addition, statistics were calculated for disease (middle ear, blood, and CSF) versus carriage populations as well as invasive (blood and CSF) versus non-invasive (carriage and middle ear) isolate populations.

### Complete Genome Sequencing

The chromosomal DNA of isolate 06-01-003MEF (CC199, serotype 19A) was sequenced using the Solexa paired-end sequencing platform (Illumina, San Diego, CA). Seventy-five bp reads were generated, resulting in an on average coverage of 16X. The paired-end sequences were trimmed based on quality score by bTrim (Kong, unpublished, http://graphics.med.yale.edu/trim/readme). Sequences that passed trimming were assembled by Velvet [53]. Different parameters of bTrim and Velvet were evaluated to optimize the final contigs with respect to n50 and the greatest contig length. This Whole Genome Shotgun project has been deposited at DDBJ/EMBL/GenBank under the accession AEGF00000000. The version described in this paper is the first version, AEGF01000000.

The genome sequence of 06-01-003MEF was compared to the publicly available genome of isolate CDC3059-06 (CC199, serotype 19A) (accession number NZ_ABGG00000000). Sequences unique to each genome were identified via subtractive BLAST analysis on the assembled sequence data sets. Sequences found to have a matching sequence in the corresponding genome were removed from both genomes. Genes were subsequently identified in the remaining unique sequences using Glimmer3 [54]. Sequence similarity with publicly available sequences was assessed using the NCBI BLAST database (http://www.ncbi.nlm.nih.gov/BLAST).

## Supporting Information

Table S1 Genes found to be variably present within CC199.
(XLS)

Table S2 Primer sequences used in the validation of CGH data.
(XLS)

Table S3 Primer sequences used to test for the presence/absence of tissue-specific genes identified by correspondence analysis.
(XLS)
